# Phylogenetic and Biological Significance of Evolutionary Elements from Metazoan Mitochondrial Genomes

**DOI:** 10.1371/journal.pone.0084330

**Published:** 2014-01-20

**Authors:** Jianbo Yuan, Qingming Zhu, Bin Liu

**Affiliations:** 1 Center of Systematic Genomics, Xinjiang Institute of Ecology and Geography, Chinese Academy of Sciences, Urumqi, Xinjiang, China; 2 CAS Key Laboratory of Experimental Marine Biology, Institute of Oceanology, Chinese Academy of Sciences, Qingdao, Shandong, China; 3 Graduate University of Chinese Academy of Sciences, Beijing, China; 4 GenomeSense Institute, Beijing, China; The Centre for Research and Technology - Hellas, Greece

## Abstract

The evolutionary history of living species is usually inferred through the phylogenetic analysis of molecular and morphological information using various mathematical models. New challenges in phylogenetic analysis are centered mostly on the search for accurate and efficient methods to handle the huge amounts of sequence data generated from newer genome sequencing. The next major challenge is the determination of relationships between the evolution of structural elements and their functional implementation, which is largely ignored in previous analyses. Here, we described the discovery of structural elements in metazoan mitochondrial genomes, termed key K-strings, that can serve as a basis for phylogenetic tree construction. Although comprising only a small fraction (0.73%) of all K-strings, these key K-strings are pivotal to the tree construction because they allow for a significant reduction in the computational time required to construct phylogenetic trees, and more importantly, they make significant improvement to the results of phylogenetic inference. The trees constructed from the key K-strings were consistent overall to our current view of metazoan phylogeny and exhibited a more rational topology than the trees constructed by using other conventional methods. Surprisingly, the key K-strings tended to accumulate in the conserved regions of the original sequences, which were most likely due to strong selection pressure. Furthermore, the special structural features of the key K-strings should have some potential applications in the study of the structures and functions relationship of proteins and in the determination of evolutionary trajectory of species. The novelty and potential importance of key K-strings lead us to believe that they are essential evolutionary elements. As such, they may play important roles in the process of species evolution and their physical existence. Further studies could lead to discoveries regarding the relationship between evolution and processes of speciation.

## Introduction

Currently, the majority of molecular phylogenetic analyses rely on sequence comparison [1], [2], e.g., the comparison of orthologous genes or of whole-genome/proteome sequences [3]–[5]. These methods can be further subdivided into two types: alignment-based approaches [6], [7] and alignment-free approaches [8], [9]. As classical phylogenetic and taxonomic methods, alignment-based approaches have been widely used in various analyses of species phylogeny [10]–[12]. However, recent advances in genome sequencing technology have facilitated the acquisition of exponentially increasing amounts of sequence data derived from individual genes and whole genomes. As a result, alignment-based methods have become less applicable due to their limited phylogenetic information potential [13], [14]. Thus, alignment-free approaches based on whole-genome/proteome sequences may provide more robust information for phylogenetic analysis [13]–[15]. Several alignment-free methods have been implemented for inferring the phylogeny of organisms: tetranucleotide-based patterns [16], singular value decomposition (SVD) [9], [17], feature frequency profiles (FFP) [18], [19] and the Composition Vector Tree method (CVTree) [20], [21]. These methods, which are based on whole-genome comparison, have been widely and successfully applied to phylogenetic analysis because of their convenience and efficiency: these methods do not require the accumulation of homologous genes for alignment and can be used to construct phylogenetic trees with superior topologies [18], [20], [22].

Even when using alignment-free approaches, handling the vast computational demands arising from whole-genome information still poses an important challenge, especially for eukaryotic species [9], [17]. Fortunately, applying analyses based on the mitochondrial genome (mt genome) presents a solution to this computational problem because of the mt genome's small size and easy isolation. The mitochondrion has its own independent genome that co-evolved with the nuclear genome during their long, symbiotic evolutionary history [23], [24]. This fact indicates that mitochondrial evolution can consistently represent the evolutionary trajectory of the host organism and can thus be used for phylogenetic analysis. A growing number of phylogenetic analyses were based on mt genome. So far, no attempts have been made to measure phylogenetic relationships among mt genomes via the CVTree method [25], [26].

The CVTree method has been successfully implemented for plant phylogenetic analysis based on whole chloroplast proteomes [27] and for prokaryote phylogenetic analysis based on whole prokaryote proteomes [20]. CVTree infers phylogenetic relationships among organisms based on the oligopeptide contents (namely K-strings) of protein sequences or from the oligonucleotide contents of DNA sequences [21]. Because CVTree utilizes calculations involving a large dataset containing either 20^K^ (for protein sequences, K is the length of K-strings) or 4^K^ (for DNA sequences) K-strings for each organism, it consumes an enormous amount of processor time and memory [28]. It appears that not all K-strings are effective for the construction of phylogenetic trees. Hence, we advance our first hypothesis: only a subset of K-strings contributes significantly to tree construction, and this subset contains sufficient information for a phylogenetic analysis relying on these strings alone. Additionally, as dimension reduction improves prediction performance [29], the use of these key K-strings should lead to the construction of more rational phylogenetic trees. Therefore, we are interested in identifying and collecting these special K-strings.

The evolutionary history of living species can be inferred via the phylogenetic analysis of molecular and morphological information using various mathematical models [30], [31]. The next round of challenges in phylogenetic analysis centers on the relationship between the evolution of structural elements and their functional implementation, a relationship that is largely ignored in the most of the analyses so far [32]. Beyond identifying the K-strings that are most important for tree construction, we are interested in analyzing their characteristics and properties, as we may consider them to be evolutionary structural elements. Phylogenetic trees can serve as the foundation for an “evolutionary synthetic biology,” which helps us to better understand the evolution of cellular pathways, macromolecular machines and other emergent properties of early life [32]. Yet, the question remains: what is the functional implementaton of a phylogenetic tree constructed from genome/proteome sequences? Furthermore, how do these structural elements support the evolution of protein structure and function? It appears that we still do not fully understand the biological/functional significance of phylogenetic trees, which reflect evolutionary relationships. As a result, we advance another hypothesis: the K-strings that contribute most significantly to tree construction contain important information regarding to the biological/functional significance of phylogenetic trees.

In this study, we performed dimensional reduction on a collection of high-dimensional data (taking K = 5, yielding 20^5^ protein strings) and obtained a cluster of key K-strings from a dataset of the whole mt genome sequences of all metazoans available. Using these key K-strings, we reconstructed the metazoan phylogenetic tree, which we then compared with the tree constructed using the CVTree method and with trees constructed using many other methods [9], [33]. We also implemented further structural analyses of these K-strings to determine the distribution pattern of amino acid compositions. We then analyzed the genetic characters of conservativity, hydrophobicity, and the special motif structure of these key K-strings. Finally, we attempted to deduce the potential biological implication of these key K-strings.

## Materials and Methods

### Dataset 1. The dataset for the extraction of key K-strings

We obtained whole-mt genome metazoan protein sequences from the NCBI web site (www.ncbi.nlm.nih.gov). In general, the metazoan mitochondrion comprises 13 protein-coding genes: ATP synthase subunits 6 and 8 (atp 6, 8), three cytochrome oxidase subunits (cox1–3), NADH dehydrogenase subunits 1–6 and 4L (nad1–6 and nad4L) and cytochrome b (cob). A total of 1,665 metazoan mt genome sequences are available online, and these organisms are classified into different groups at the phylum level (Table S1). Twenty-four variable phyla (including subphyla) are present and were considered for further analysis.

### Dataset 2. The dataset for the tree construction

After collecting key K-strings, we randomly selected 87 species for the construction and comparison of phylogenetic trees (Table S2). In each phylum four or less species were selected, except in Vertebrata, for which we selected five species from each class because of the multitude of species in each class. We randomly selected species from other phyla with data available for more than four species. In the comparative study to the SVD tree [9] and the other trees [33], the datasets were taken from each corresponding references.

### CVTree algorithm

The CVTree method [20], which has been updated since its introduction [28], is mainly based on a (K-2) Markov model. This method entails counting the frequencies of the 20^K^ types of K-strings (K = 3 to 7, instead of the length of short-sequence strings) subtracted from a mutational background obtained from a K-2 Markov model. Next, a phylogenetic tree is constructed based on a cosine distance matrix. As K is a number ranging from 3 to 7, the compositional vector (CV) matrix has 20^K^ columns representing the 20^K^ strings generated from protein sequences. Previous studies have indicated that trees constructed for K = 5 are superior to those constructed with other values of K [20], [34]. In light of this fact, we chose K = 5 for the analysis of 20^5^ (3,200,000) K-strings. Then, two types of key K-strings are collected: (i) broad key K-strings, which contribute significantly to the classification of species in different phyla; and (ii) phylum-specific key K-strings, which describe the phylogenetic relationships among species below the phylum level.

### Dimensional reduction

**Figure 1:**
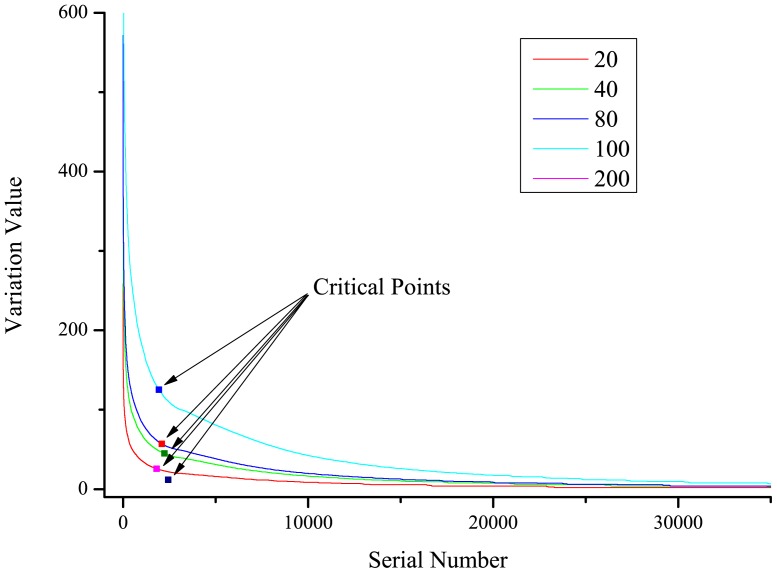
Critical points of the L-curve. The critical point represents a 90% reduction in the maximum variation value, and points above the critical point correspond to key K-strings. The curves were generated based on randomly selected datasets with different numbers of species, (20, 40, 80, 100, or 200). Points with serial numbers greater than 35,000 do not appear under zoom.

As for a group of protein sequences from mt genomes, CVTree can be used to obtain a CV matrix encoding the total phylogenetic information for this group. To collect the key K-strings, we implemented a dimensional reduction on this CV matrix. We calculated the variance *D(X)* for each column of the matrix to stand for the variation value of every dimension that contributes to the construction of a tree:
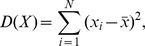



N is the number of rows in the matrix, which stands for the number of species analyzed. 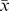 is the mean value of each column of the matrix, namely, each K-string. We sorted these 20^K^ variation values and placed them into a scatterplot, which is L-shaped (Figure 1). We then identified a critical point dividing these points into two groups that contribute either greatly or slightly to tree construction. The L-shaped corner has generally been considered to be the best corner [35], [36]. The critical point, which is located at the corner of the L-type curve, is identified by a 90% reduction of the maximum variation value. The points with variation values larger than the critical point serve as our key points, and the corresponding K-strings yield the key K-strings.

### Extraction of the key K-strings

Considering that more than half of metazoan species are vertebrates, we first took Vertebrata as an example for a detailed analysis. We randomly selected twenty different species as the test group and obtained a cluster of key K-strings through dimensional reduction. We then repeated this procedure until the newly generated key K-strings were almost totally represented in our existing set of key K-strings. Finally, we formed the intersection and union of these key K-strings. One may view these key K-strings as broad key K-strings. As with the class-specific key K-strings, we followed the above procedures inside each class. In order to collect the total key K-strings of metazoan, the broad and phylum-specific key K-strings were also extracted from all the metazoans following the above procedures. Hence, a union of these key K-strings was collected to yield the global key K-strings for metazoans.

### Tree construction and comparison

We constructed key K-string trees based on cosine distance matrices that we obtained by calculating the cosine value (c) of every two vectors in the CV matrix:
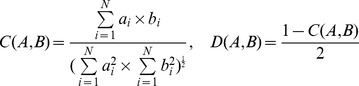



*C(A, B)* is the correlation between two species A and B, while *D(A, B)* stand for the distance between the two species. a_i_ and b_i_ is the vector of the N-dimensional space of two species A and B.

The neighbor-joining methods [37] algorithm in the PHYLIP [38] software tool was used for the distance-based tree construction, and the visualization of the trees was implemented in MEGA software [39], which render trees without any consideration of branch lengths so that the tree topology can be clearly displayed. In addition, a bootstrap test was implemented to get branch support values [40]. In doing bootstrap test, protein sequences were picked up randomly from the pool of all mitochondrial proteins of a species. Some protein sequences would be drawn repeatedly, while others might be skipped. On average, about 80% of protein sequences were kept with some repetitions and the total length of protein sequences will not be changed at each calculation. Then, 100 trees can be obtained from 100 bootstrap replicates, and the bootstrap values can be produced by CONSENSUS program in the PHYLIP package.

We applied tree comparison to four trees: (i) a tree constructed from our global key K-strings (the key K-strings tree); (ii) a corresponding tree constructed from the same number of randomly selected K-strings, serving as a control (the normal tree); and (iii) a tree constructed from the complete set of 20^5^ K-strings using the CVTree method (the CV-tree). and (iv) an alignment-based tree constructed by Phyml using maximum likelihood analysis (ML tree) [41]. For the alignment-based tree, all the mitochondrial protein sequences were completely aligned by MUSCLE 3.6 [42], and the conserved region of each alignment was trimmed using Gblocks [43], which allows less strict flanking positions and gap positions within the final blocks, but does not allow many contiguous nonconserved positions. Then, ML analysis was implemented on the alignment with an model of JTT + gamma, and 1000 bootstraps were performed to gain the branch support values. Furthermore, we also compared our key K-string trees with the trees constructed by Yu Zuguo [33] and those constructed using the SVD method [9]. A robust wed-based tool were implemented for comparing these phylogenetic trees [44], and a score of overall topological similarity can be calculated between any two trees.

### Compositional analysis of the key K-strings

We applied a compositional analysis to determine the composition patterns of the key K-strings. We calculated single amino acid compositions in addition to both contiguous and interval dimer (i.e., triplet) compositions for broad and phylum-specific key K-string groups and for the original entire protein sequences.

### Conservativity analysis of the key K-strings

To investigate the biological implications of our key K-strings, we mapped them back onto the original protein sequences. We identified 400 broad key K-strings in Vertebrata that are the most important for distinguishing species belonging to different classes. After selecting 10 species at random from vertebrates, with 2 species included per class, we performed a complete alignment of these protein sequences using the ClustalX software [45]. We calculated the conservativity distribution pattern by sliding across sequences with a window size of 5 aa and step size of 1 aa. As a result, the locations of the 400 key K-strings were clearly displayed on the protein sequences. Thus, we can compare the areas in which key K-strings accumulated to conserved regions of the protein sequences to reveal relationships among them.

### Statistical methods

We implemented an adaptive chi-square test to verify the existence of significant differences between the observed and predicted frequencies of K-strings. The observed frequencies of a 5-string (K = 5) are given by *p*(a_1_a_2_a_3_a_4_a_5_); two corresponding 4-strings are *p*(a_1_a_2_a_3_a_4_) and *p*(a_2_a_3_a_4_a_5_), and one 3-string is *p*(a_2_a_3_a_4_). The predicted theoretical value of the 5-string is denoted *p* ^0^(a_1_a_2_a_3_a_4_a_5_), and its chi-square test value is denoted *X* ^2^:
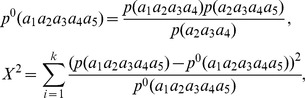



Here, k stands for the number of K-strings tested. We examined differences at the 5% significance level in the chi-square test.

## Results

In this study, one of the most important steps was dimensional reduction to yield a 90% reduction of the maximum variation value, the critical value for separating key K-strings from the remaining K-strings. After sorting variation values and plotting them against the series number, an L-shaped curve with no inflection point emerged (Figure 1). Therefore, the critical point, which was located at the corner of the curve, divided the points into two groups: those having obvious large and small variation values. We determined the locations of critical points with different group sizes (20, 40, 80, 100 and 200 species). As shown in Figure 1, almost all of the critical points in each group were located at the corner of the L-shaped curve, suggesting that we can ignore points found below the critical point. Thus, points located above the critical point were putatively identified as our desired key points, i.e., those that should contribute significantly to tree construction in distance-based phylogenetic analyses. Therefore, we considered the corresponding K-strings of these key points to be key K-strings.

### Broad key K-strings

In the vertebrates, we obtained 10 groups of broad key K-strings by repeating the above procedure 10 times. In each group of species, all the species are equally divided into 5 subgroups. To ensure the quality of these key K-strings, we collected each group of broad key K-strings from the intersection of 5 clusters of key K-strings generated from 5 subgroups. The size of the 10 groups of broad key K-strings ranged from 656 to 837, with a mean value of 750. Finally, we formed convergence intersection and union sets of the 10 groups of broad key K-strings, containing 400 and 1211 K-strings, respectively. Thus, these 400 K-strings were essential for any tree construction over vertebrates because they appeared in every group of broad key K-strings, and the 1211 K-strings from the union set contained all the broad key K-strings from 10 groups. Through using these 400 and 1211 K-strings to construct phylogenetic trees, we found that the resulting trees successfully classified five classes of Vertebrata but failed to describe the phylogenetic relationships of the species in each class when compared with the trees constructed by the CVTree method. Therefore, although these two clusters of key K-strings did not contain enough information for tree construction, they still reflected the phylogenetic relationships among vertebrates at the class level, so we considered them to be our broad key K-strings.

**Figure 2:**
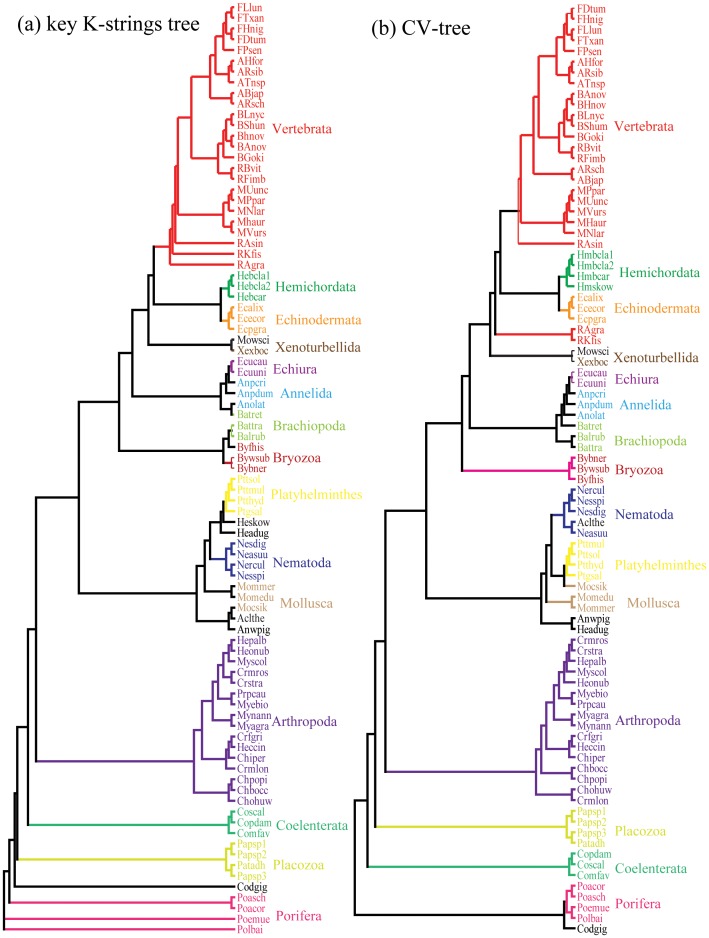
Phylogenetic trees constructed from our key K-strings and complete K-strings using the CVTree method. Both trees contain 87 species that were randomly selected from each phylum (Table S2). (a) The phylogenetic trees constructed from our 23,223 key K-strings; (b) The phylogenetic trees constructed from the complete set of 20^5^ K-strings using the CVTree method. Bootstrap support values above 40% from 100 replicates were show in the figure.

As with the broad key K-string analysis in Vertebrata, we obtained 2,552 broad key K-strings of metazoans when setting the 87 species from dataset 2 as our test group. As expected, the phylogenetic tree constructed from these broad key K-strings exhibited a topology similar to that of the tree formed with the CVTree method (Figure 2.b), but on comparison with the CV-tree, we observed that 5 species were differently placed.

### Phylum-specific key K-strings

In general, document classification [46] is considered to be similar to alignment-free phylogenetic analysis because both methods calculate the frequencies of the basic elements appearing in the complete documents/sequences and classify different articles/species into different groups. Just as different types of articles have different style specifications, different phyla may also have their own specific key K-strings. Therefore, one may extract phylum-specific key K-strings from the datasets of species belonging to each phylum. As with the five classes of Vertebrata, we obtained five clusters of class-specific key K-strings and grouped them into 1211 broad key K-strings. Finally, we obtained a union set of 3,055 K-strings, which we considered to be the phylum-specific key K-strings of vertebrates. Similarly, we collected the phylum-specific key K-strings of metazoans by applying the methods above (Table 1). We found that the number of phylum-specific key K-strings ranged from 1,956 (for Mollusca) to 4,656 (for Annelida) with a mean number of 3,248. However, the presence of shared key K-strings between any two species was rare; only 193 key K-strings were shared between Coelenterata and Nematoda, and the mean number of shared key K-strings was 510 among all phyla. Thus, a significant bias existed for the usage of phylum-specific key K-strings between any two phyla, as illustrated in the lower triangular matrix of Table 1. The frequencies of differences ranged from 60.5% (between Crustacea and Hexapoda) to 94.9% (between Platyhelminthes and the vertebrates), with a mean value of 83.6%. In addition, phylogenetically closely related phyla shared more specific key K-strings, whereas distant phyla shared fewer strings. Crustacea and Hexapoda, both in the subphylum Arthropoda, shared the most specific key K-strings, with 1,117 in common, whereas Platyhelminthes and Vertebrata, which are located far from each other in the traditional phylogenetic tree [47], [48], had the largest bias percentage, at 94.9%, and shared only 223 specific key K-strings. Therefore, phylum-specific key K-strings were mostly unique to their corresponding phylum and were tightly correlated with phylogenetic distance. We arranged all of the phylum-specific key K-strings and the broad metazoan key K-strings into a group containing a total of 23,223 K-strings. Surprisingly, these 23,223 key K-strings constituted only a small fraction (0.73%) of all the 20^5^ K-strings.

**Table 1:** The number of phylum-specific key K-strings and their diversity.^a^

	Annelida	Chelicerata	Coelenterata	Crustacea	Echinodermata	Hexapoda	Mollusca	Nematoda	Platyhelminthes	Porifera	Vertebrate
**Annelida**	**4656**	721	308	748	669	782	641	330	367	510	611
**Chelicerata**	84.5	**2987**	349	911	718	1091	611	534	579	559	511
**Coelenterata**	91.8	88.3	**2642**	308	384	328	274	193	259	777	291
**Crustacea**	83.9	69.5	88.3	**2827**	674	1117	612	342	438	500	552
**Echinodermata**	85.6	76	85.5	71.2	**3340**	682	491	343	503	594	685
**Hexapoda**	83.2	63.5	87.6	60.5	79.6	**3197**	559	454	464	549	576
**Mollusca**	86.2	79.5	89.6	78.4	85.3	82.5	**1956**	291	345	418	428
**Nematoda**	92.9	82.1	92.7	87.9	89.7	85.8	85.1	**2545**	485	309	195
**Platyhelminthes**	92.1	80.6	90.2	84.5	84.9	85.5	82.4	80.9	**4326**	448	223
**Porifera**	89.1	81.3	70.6	82.3	82.2	82.8	78.6	87.9	89.6	**4198**	435
**Vertebrate**	86.9	82.9	89	80.5	79.5	82	78.1	92.3	94.9	89.6	**3055**

^a^ The thick, black collection of data points along the diagonal represents phylum-specific key K-strings belonging to different phyla. The upper triangular matrix encodes the key K-strings numbers shared by both phyla. The lower triangular matrix provides the percentage of bias represented by key K-strings belonging to two different phyla.

### Key K-strings successfully used for tree construction within metazoa

To determine whether these 23,223 key K-strings are essential for phylogenetic tree construction for the metazoans, we constructed a tree based on these key K-strings (Figure 2.a) and compared it with the corresponding tree for normal K-strings and the CV-tree (Figure 2.b). We found that the normal K-string tree, which we constructed from 23,223 randomly selected K-strings, did not group species belonging to the same phylum and did not accurately describe the phylogenetic relationships among the phyla of the metazoa, suggesting that a random collection of K-strings is not useful for tree construction. With the help of the web-based tool for pairwise phylogenetic trees comparison [44], we found that our key K-strings tree and the CV-tree are highly similar in tree topology (overall topological score of 84.7%). From the results of tree comparison, fewer differences were observed aamong them; only two phyla, Coelenterata and Placozoa, exchanged their phylogenetic location. Previous researches indicated that Coelenterata developed the specialized nerve cells when comparing with Placozoa and Porifera, which possessed a pre-nervous system [49]. It was also supported by a maximum likelihood phylogenetic tree which agrees with our key K-strings tree [49]. When comparing both trees, except several species, almost all the species were successfully grouped within each phylum. These exceptional species were both showed similar phylogenetic location on two trees. However, there were also differences between two trees that two reptiles, *Acrochordus granulatus* (RAgra) and *Kinyongia fischeri* (RKfis), were not grouped with other vertebrates in the CV-tree, but they were correctly located in the key K-strings tree. When analyzing the deep phylogenetic relationships among the 25 species in Vertebrata beneath the phylum level, these two trees exhibited similar topologies except for two Amphibians: *Bufo japonicus* (ABjap) and *Rhacophorus schlegelii* (ARsch). In the CV-tree, these two Amphibians were not grouped with the other three, whereas they are accurately grouped with other Amphibians in key K-strings tree. Therefore, the phylogenetic tree constructed from all 23,223 key K-strings can accurately describes the phylogenetic relationships among the metazoans as CVTree, even beneath the phylum level; in fact, our key K-strings trees are somewhat superior to those derived from CVTree.

Alignment-based phylogenetic analysis are generally used in previous researches [6], [7], and we also constructed a ML tree based on the alignment of all the mitochondrial protein sequences (Figure S1). Both key K-strings tree and CV-tree showed highly similarity to this ML tree (the topological scores between K-strings tree, CV-tree and ML tree are 75.3% and 73.7%, respectively). Although statistically similar, there are some differences between key K-strings tree and ML tree. Like that of CV-tree, ML tree placed branch of Coelenterata behind Placozoa, which is just on the contrary to key K-strings tree. Furthermore, in the branch of Vertebrata, the phylogentic location of Birds and Mammalians are exchanged between ML tree and the two alignment-free trees. Besides, several other differences also have been detected between key K-strings tree and ML tree, especially in the branch of Arthropoda, whose phylogeny has not been illustrated clearly at present time. It seems the overall performance of key K-strings tree is similar to that of alignment-based tree, although some differences exist.

In the traditional view of metazoan phylogeny [47], [48], Protostomia, which include Arthropoda, Annelida and Mollusca, are sister groups to Nematoda or Porifera, whereas Deuterostomia (Chordata, Hemichordata, Echinodermata and Brachiopoda) have remained stable as a monophyletic group [47]. However, as metazoan phylogenetic analysis has progressed, many new metazoan phylogenies have been published [47], [48] that grouped Arthropoda and Nematoda as Ecdysozoa and also grouped Mollusca, Brachiopoda, Annelida and Platyhelminthes as Lophotrochozoa, having evolved from Porifera. It became evident that Deuterostomia in the key K-string tree are phylogenetically distant from Porifera and also that some differences exist between the key K-string tree and these other trees. The key K-string tree showed a close relationship among Platyhelminthes, Mollusca and Nematoda, three phyla of “worms” [50], [51]. In contrast, Annelida was excluded from this group and was instead lumped with Brachiopoda, Bryozoa, and Echiura, similarly to the phylogenetic tree of Shinichi Yokobori [52]. The phylogenetic position of the phylum Arthropoda as a member of the Protostomes is controversial [53], [54]. The classical hypothesis holds that Annelida is the closest phylum to Arthropoda [55], whereas the Eutrochozoa hypothesis alternatively establishes that Nematoda is the closest [56]. However, in our work, Arthropoda is a monophyletic group constituting a sister group to both Placozoa and Porifera and also showing a close relationship with Nematoda and Platyhelminthes.

In addition to studying traditional alignment-based trees, we also compared our key K-strings tree to other alignment-free trees such as the SVD tree [9] and the three trees constructed by Yu Zuguo [33]: the dynamical language model with correlation distance (DLM) tree [27], the Fourier transform with Kullback-Leibler divergence distance (KLD) tree [57] and the log-correlation distance (LCD) tree [17]. In the first study, Yu indicated that the phylogenetic tree generated by the CVTree method did not clearly separate fish, birds and reptiles [33]. However, few divergences from the three trees of Yu were evident in our key K-strings tree (Figure S2). When comparing our tree to the tree constructed using KLD distance, only two different arrangements were present: *Falco peregrinus* (Fper) and *Danio rerio* (Drer). When using the DLM approach, only one species (*Smithornis sharpei* (Ssha)) segregated differently, and three species, *Corvus frugilegus* (Cfru), *Falco peregrinus* (Fper) and *Smithornis sharpei* (Ssha), were located differently when using the LCD method. Furthermore, the key K-string tree was somewhat more intuitive than other methods regarding its accuracy in that every species was grouped within its corresponding phylogroup. In contrast, the KLD method arranged the Cartilaginous Fish as a subgroup of the Bony Fish; the DLM approach put *Protopterus dolloi* (Pdol) into the phylogroup of the Cartilaginous Fish; and the LCD method placed *Sus scrofa* (Sscr) into Carnivores and *Protopterus dolloi* (Pdol) into Cartilaginous Fish. Overall, we effectively used the key K-strings identified for the phylogenetic analysis of metazoans, and the trees constructed from these 23,223 key K-strings appeared to exhibit a more rational or even superior topology to the CV-tree and many other trees. Thus, our results supported our first hypothesis that only a fraction of the K-strings contribute significantly to tree construction, and these strings contain sufficient information for phylogenetic analysis.

### Composition patterns vary among different groups of key K-strings

**Figure 3:**
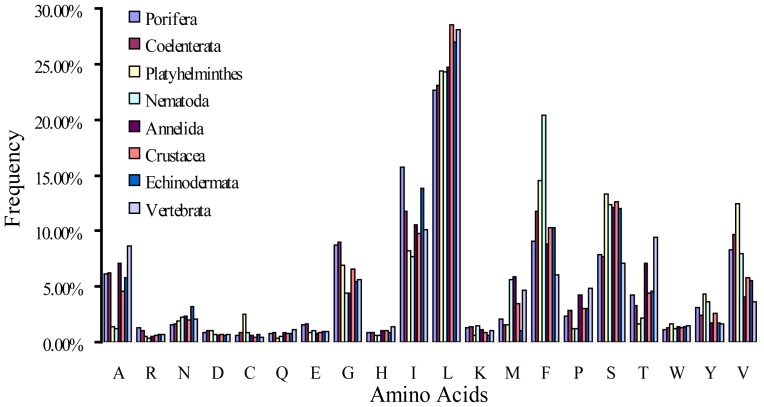
Single amino acid compositions of the key K-strings in each phylum. The compositional patterns of 8 groups of phylum-specific key K-strings are illustrated using different colors.

Considering that key K-strings are closely associated with phylogeny, the analysis of their compositional patterns was of interest. As for the overall set of key K-strings, ten amino acids, viz., A, G, I, L, M, F, P, S, T, and V, are used more frequently than the others, and the majority are apolar amino acids. We found a similar distribution pattern in 8 groups of phylum-specific key K-strings (Figure 3). Several differences also existed in that Nematoda and Platyhelminthes used lower quantities of A, I, P and T and higher quantities of F than other phyla; Platyhelminthes used notably more C than the others, and the amino acid M was predominant in Nematoda, Annelida, Crustacea and Vertebrata.

**Figure 4:**
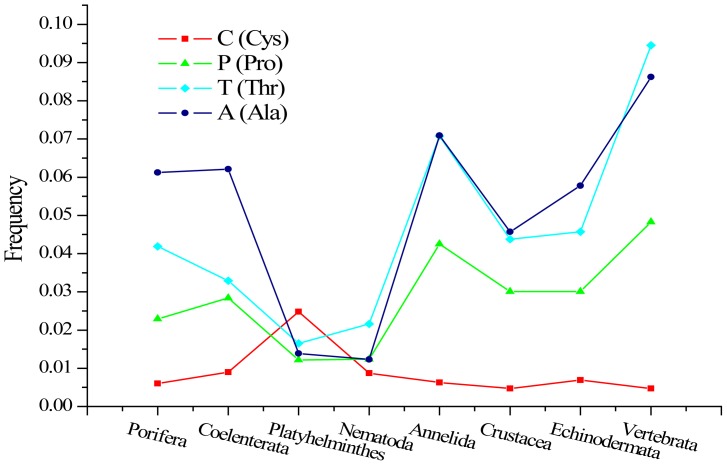
The composition patterns of phylum-specific key K-strings in different groups. The 8 phyla are arranged according to the traditional view of phylogeny: Porifera, Coelenterata, Platyhelminthes, Nematoda, Annelida, Crustacea, Echinodermata and Vertebrata. Single amino acids, dimers and triplets containing the amino acids A, P or T displayed one distribution pattern, whereas those containing C exhibited a different distribution pattern.

In addition to single amino acids, we also analyzed the compositions of dimers and triplets in the key K-strings. We found that almost all the dimers and triplets containing A, P, and T or C had similar distribution patterns resembling the distribution of single amino acids (Figure S3, Figure 4). The distribution patterns of A, P and T displayed two troughs, and only one peak appeared in the distribution pattern of C. It is generally known that cysteine (C) is the key amino acid for protein structure, forming strong bridging (disulfide) bonds. Furthermore, alanine (A) is frequently used to design and construct diverse, well-defined three-dimensional structures [58], and proline (P) is preferred for forming β-sheets and random coils at the corner of a protein, thereby enhancing the stability of the protein's spatial structure [59], [60]. Therefore, the selected usage of the amino acids, dimers and triplets contained in the key K-strings might be linked to the formation of various higher protein structures in different species.

### Key K-strings located substantially within conserved regions of protein sequences

**Figure 5:**
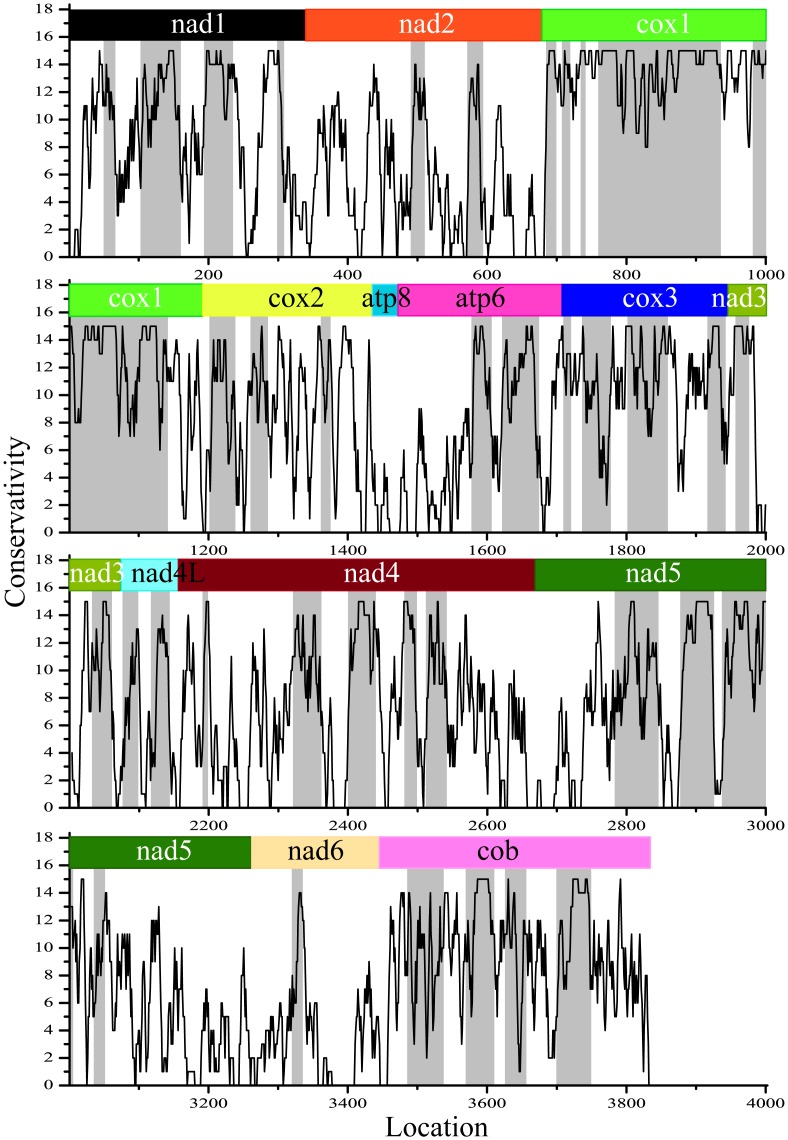
Distribution of conserved regions and regions in which key K-strings accumulated for complete protein sequences. Thirteen protein sequences are aligned in order and linked head-to-tail for the 10 chosen vertebrates. The curve models the distribution of sequence conservativity, and gray regions represent regions in which key K-strings accumulate.

Considering the fact that the key K-strings were small fractions in the complete protein sequences, an intriguing problem is to determine where they are located and whether they are associated with special protein characters or properties. We analyzed several characters and properties of the parent protein sequences (conservativity, hydrophobicity, functional motifs and active sites), but we found no obvious association between these characters and the key K-strings except for conservativity. We mapped 400 broad key K-strings in Vertebrata to the corresponding full-length protein sequences and analyzed their global location. We were surprised to observe that these key K-strings tended to accumulate at different regions of the protein sequences, which we named accumulation regions (Figure 5). With the help of ClustalX software [45], we obtained the conservativity distribution pattern of protein sequences for vertebrates and located highly conserved regions (see the “Methods” section). The conservativity values ranged from 0 to 15, where a value of 15 indicates that a locus is highly conserved, i.e., every segment in the different sequences is the same at this position. In contrast, a conservativity value approaching 0 indicates strong variation at the associated locus. We were rather surprised to observe that the key K-string accumulation regions appear to mainly coincide with the conserved regions in the protein sequences (Figure 5). The majority of the key K-string accumulation regions were highly conserved, having conservativity values greater than 8. Therefore, we may reasonably assume that the resulting phylogenetic trees are constructed from highly conserved regions of protein sequences.

## Discussion

### Dimensional reduction superior in the phylogenetic tree from CVTree

Dimensional reduction is the process of reducing the number of variables under consideration, a process that can be classified as either feature selection or feature extraction [61], [62]. Feature selection was used in this study, i.e., the selection of a subset of the relevant features under consideration. Feature selection provides many potential benefits: facilitating data visualization and data interpretation, reducing measurement and storage requirements in computation, reducing training and time utilization, and addressing the problem of dimensionality to improve prediction performance [29]. We performed feature selection by selecting points with variation values greater than 90% of the maximum reduction and obtained 23,223 key K-strings, which is only a small fraction (0.73%) of all the possible 20^5^ K-strings. Using this dimensional reduction, we easily performed a composition analysis, a conservativity analysis and a biological significance analysis on these key K-strings. Additionally, considering that the key K-strings only composed 0.73% of all K-strings, we drastically reduced the runtime (by a factor of approximately 100) required to calculate the CV matrix, which makes the phylogenetic analysis of larger datasets a feasible task that would be easy enough to perform even on a notebook computer. Most importantly, dimensional reduction can improve the prediction performance of a phylogenetic tree; the tree constructed from the key K-strings exhibited a more rational topology than the CV-tree and many other alignment-based or alignment-free trees [33], [63]. In many other feature selection studies, irrelevant or redundant candidate features are present that do not affect the target concept; these features sometimes even act as noise, degrading predictions and increasing computation time [64]–[67]. Therefore, one may consider key K-strings to be relevant features that are essential for tree construction and view other K-strings as irrelevant or redundant features that are useless for tree construction and may even degrade the tree's topology. The purpose of dimensional reduction is to obtain the smallest possible number of features that adequately represent the complete set of information for the data and then maximize prediction or classification accuracy [68], [69]. Thus, we may reasonably assume that although a great many K-strings were removed, the key K-strings still contained adequate phylogenetic information for tree construction. Above all, dimensional reduction can facilitate an understanding of these phylogeny-related key K-strings and improve the performance of phylogenetic tree construction and analysis.

Although dimensional reduction is benefitial to phylogenetic tree construction, there are still some limitations of the approach taken in this study. Firstly, from current available metazoans mitochondrial genomes, the groups of key K-strings obtained currently provide a base view the metazoan phylogenetic analysis more refinement is needed when when more mitochondrial genome are sequenced. We did a test on this approach, and we found that if 100 vertebrates added for the analysis, the overall key K-strings of vertebrata are changed slightly (94 key K-strings attended on average). But when we added a new phylum for the analysis, there are average 1646 key K-strings will be attended in the final group of key K-strings. Therefore, although a full list of mitochondrial genomes of metazoans are yet fully sequenced, the group of key K-strings may not fluctuate significantly unless a new phylum of metazoa is detected and sequenced. Secondly, the approach can be easily performed on mitochondrial genome, but it may be more difficult for the analysis on nuclear genome or other complex genetic materials. In this study, key K-strings tend to accumulated in the conserved regions of mitochondrial protein sequences. Whereas, nuclear genome contains a much greater numbers of genes and most of genes are specific for each species and are more divergent. Therefore, it seems uncertain how well does this approach perform on nuclear genome. However,as for CVTree methods have been successfully implemented on whole fungi genome and prokaryote genome [25], [70], the dimensional reduction of this study may also be effectively used in the analysis of nuclear genome. Lastly, like other alignment-free methods, this approach does not use any model of evolution. The traditional phylogenetic methods generally use a model of evolution to correct for multiple substitutions on the same position, but it seem unable to do it in alignment-free methods. Nevertheless, a K-2 Markov model has been used for subtract the random background from single counting results [20]. Thus, the randomness caused by neutral mutations can also be eliminated, which is similar to the correction from a chosen model of alignment-based phylogenetic analysis.

### Different groups of key K-strings determine various evolutionary directions

Based on these results, we note that a significant discrepancy exists between phylum-specific key K-strings and broad key K-strings and between any two phylum-specific key K-string groups. Broad key K-strings may classify species into their correspondent phylogroups but do not illustrate phylogenetic relationships accurately within any phylum. To solve this problem, we can use phylum-specific key K-strings, each carrying unique phylogenetic information. Therefore, different groups of key K-strings appear to be somewhat associated with each species' evolutionary trajectory. In document classification, the highest priority is to find relevant information, particularly in electronic documents [71]. Several studies have discussed how to effectively and automatically classify documents into separate classes [72]. As with document classification, broad key K-strings have helped classify studies into different styles [46], [72], e.g., literature and natural science, whereas specific key K-strings have played different roles such as subdividing studies into different subjects of the same style, for example, classifying mathematics and biology as two divisions of natural science. Therefore, we suggest using broad key K-strings to allocate species into phyla and phylum-specific key K-strings to classify species belonging to the same phylum. If one text is written with a greater number of style-specific features, then it should be classified as a text belonging to that style [73]. Analogously, species with protein sequences containing more phylum-specific key K-strings in a single group will finally evolve into species belonging to that phylum. Previous researches indicated that the conserved structure cores of homologous proteins can modify their shape during evolution, and most importantly, these conserved cores can determine the evolutionary directions of deformation [74], [75]. As for the key K-strings and structure cores both located on the conserved regions of homologous proteins and they both contribute to the evolution of organisms, it is reasonable to consider these key K-strings also play a role like the structure cores to affect the evolutionary deformation and finally determine the evolutionary directions of the organism. Although the determination of whether these key K-strings actually affect the evolution of the species must be verified by biological experiments, the phylogeny-related key K-strings are undoubtedly associated with the analysis of evolutionary relationships. Therefore, we can reasonably hypothesize that key K-strings may be used to determine evolutionary trajectories.

### Key K-strings' connection to evolutionary selection pressure

Selective pressure is the major factor in the evolution of DNA sequences [76], [77] and is always tightly correlated with conserved domains in these sequences [78]. Due to evolutionary selective pressure, the functional features of sequences have evolved more slowly than non-functional features, and many short conserved motifs have been detected as functional regions in DNA sequences [79]–[81]. In this context, sequence conservation is considered to be caused by strong selective pressure to maintain the function of each protein, which explains the fact that sequence conservation is predominantly observed in coding regions [79]. In our observations, key K-strings mostly accumulated in highly conserved regions, suggesting that these strings are highly conserved in the sequences and may undergo evolutionary selective pressure. In previous studies, distance-based methods of phylogenetic analysis have attempted to identify sequence diversity and thus guide phylogenetic reconstruction [82]. However, the phylogenetic analysis in this study mainly relies on the conserved regions of the sequences in which key K-strings are located, whereas regions with high disparities have fewer effects on phylogenetic tree construction. It appears that species have retained these key K-strings to retain their original phylogenetic information over their long evolutionary history. Thus, these key K-strings may experience strong selective pressure and are thereby largely preserved. The observed and theoretical frequencies of a K-string are p(a_1_a_2_a_3_a_4_a_5_) and p^0^(a_1_a_2_a_3_a_4_a_5_), respectively (see the “Methods” section). The chi-square test indicated that a significant bias exists between the two frequencies and that the actual frequencies are far larger than the theoretical values. Therefore, key K-strings seem unusually selected over other (normal) K-strings, which may be the result of selective pressure.

### Biological significance of the key K-strings

Many biologically meaningful patterns such as life histories and ecological strategies are inherently structured by phylogeny [83]. Hence, many studies have attempted to find associations between phylogenetic trees and the evolution of cellular pathways and macromolecular mechanisms [32] or between phylogeny and biological traits [84]. Phylogenetic tree construction aims to accurately describe the evolutionary relationships among species by clustering species with similar phenotypes [85]. Yet, how can a phylogenetic tree determine evolutionary relationships among species? And what is the biological significance of structural elements for tree construction? This study offers the first attempt to determine the biological significance of a phylogenetic tree and reveal the essential association between a phylogenetic tree and biological features based on the physical existence of key K-strings. Our novel approach and the discovery of key K-strings and their successful application to metazoan phylogeny reconstruction indicate that key K-strings may play a pivotal role in species evolution. Following the methods in this paper, key K-strings can also be extracted from other genetic materials such as plastid/chloroplast, microbial and nuclear genomes. As a result, key K-strings could lead to discoveries about the relationships between evolution and the functionality of speciation.

Biologists generally accept that a phylogenetic tree can indicate whether a trait or phenotype shared by two species is the result of a common ancestry or whether the trait arose independently on the species' evolutionary trajectories; phylogenetic trees can serve as the foundation for an “evolutionary synthetic biology” that can help us to better understand the evolution of cellular pathways, macromolecular machines and other emergent properties of early life [32]. Phylogenetic comparative methods have considered phylogenetic data as a source of statistical bias in the correlative analysis of biological traits [84]. Therefore, phylogenetic trees may help guide organisms in forming their unique biological traits by regulating the composition of structural sequence elements. Conversely, in this study, key K-strings served as the structural elements in whole protein sequences that might functionally determine a species' evolutionary trajectory. Species evolution mainly depends on the expression level and functional properties of the corresponding proteins [86]. However, protein activity appears to be greatly affected by each protein's 2-D or higher-dimensional structure, which is in turn influenced by sequence composition [87]. Our results indicate that key K-strings possess a special amino acid composition pattern that contributes to the makeup of a protein's structure. Additionally, in previous studies, functional motifs were generally located within conserved regions of sequences that regulated protein expression and function [88], [89], which were rightly correlated with the location of key K-strings. Furthermore, a protein's conserved regions are crucially important for its function [90]-[93]. The small, highly conserved regions of biological significance regulate transcriptional transactivation and cell-cycle arrest [94], and the majority of conserved regions appear to be correlated with regulation and binding functions [93]–[95]. Therefore, key K-strings correlated with proteins' conserved regions may also serve as the essential elements in regulating protein function. Due to their novelty and potential importance, we propose that these K-strings are the potential essential elements of species evolution.

## Conclusions

With the help of dimensional reduction, we have accumulated a subset of 23,223 key K-strings that exhibited a strong relationship with species evolution. The trees constructed from the key K-strings not only decreased computation time, but also exhibited a more rational topology from CVTree and many other alignment-based or alignment-free trees. Notably, the key K-strings tend to accumulate at the conserved regions of homologous proteins and have special compositional characteristics that benefit for the deformation of proteins structure., Finally, the key K-strings has potential applications to the determination of various evolutionary trajectories. The novelty and potential importance of key K-strings lead us to believe that they are essential evolutionary elements. To our knowledge, this is the first report to discus the biological significance of these evolutionary elements on complementary protein sequences. As such, they may play important roles in the process of species evolution, and their identification may therefore lead to a new era of discoveries in regard to the relationship between evolution and the functionality of speciation. The significance of these elements may transcend the mt genome, and their future study will reveal their significance in plastid/chloroplast, microbial and nuclear genomes.

## Supporting Information

Figure S1 **ML phylogenetic tree of 87 species.**
(TIF)

Figure S2 **Phylogenetic tree of 64 vertebrate species constructed from 3,055 phylum-specific key K-strings found in Vertebrata.**
(TIF)

Figure S3 **Compositional analysis of phylum-specific key K-strings in 8 phyla.**
(TIF)

Table S1 **The dataset of metazoan species for which complete mitochondrial genomes are available.**
(DOC)

Table S2 **The dataset of 87 species used for tree construction and comparison.**
(DOC)
