# Supplementary figures and images for: Phylogenetic and Biological Significance of Evolutionary Elements from Metazoan Mitochondrial Genomes
Source: PLoS One. 2014 Jan 20;9(1):e84330. doi: 10.1371/journal.pone.0084330 (PMC3896360; doi:10.1371/journal.pone.0084330)

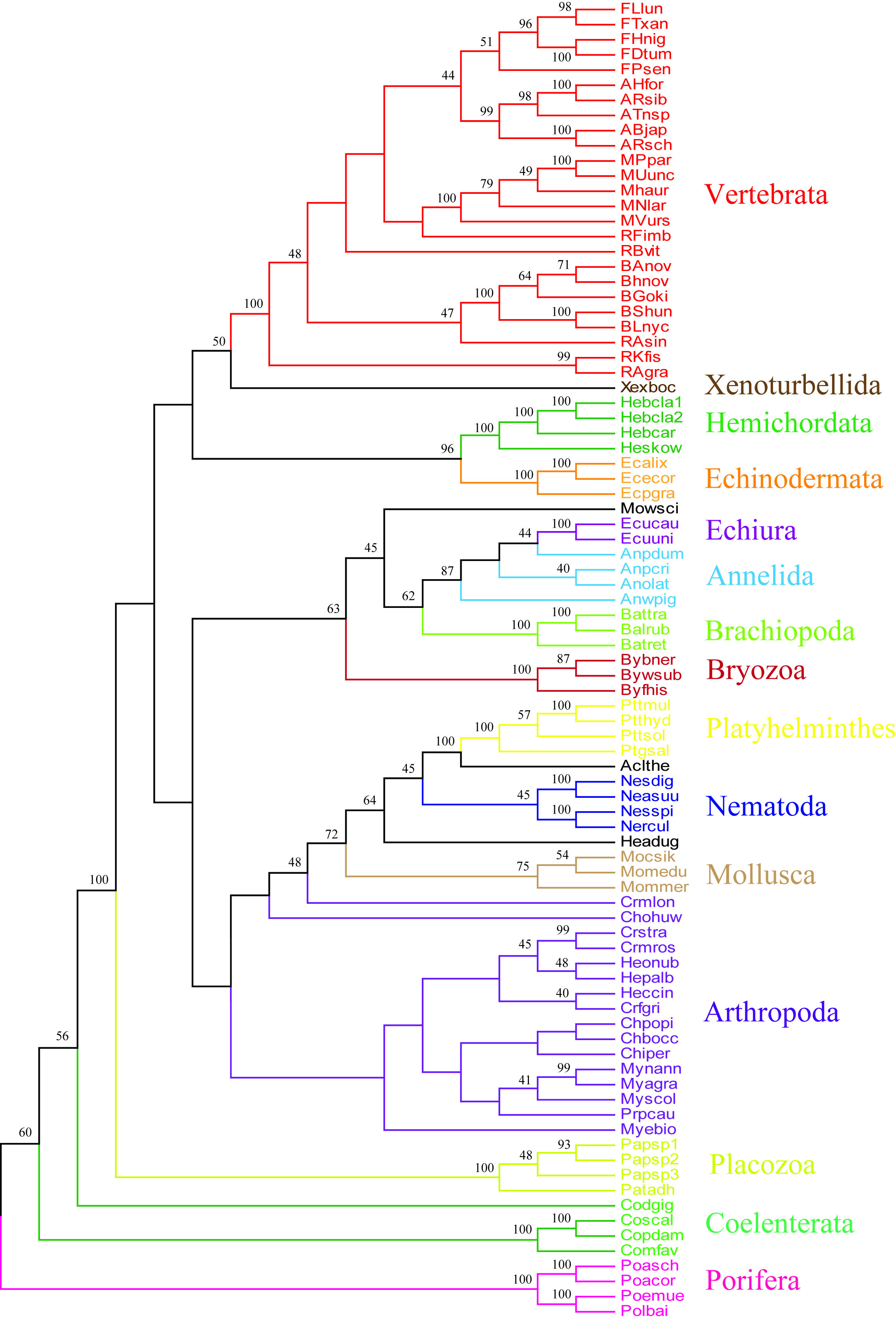

Supplement: Figure S1 — ML phylogenetic tree of 87 species. (TIF) [file pone.0084330.s001.tif]

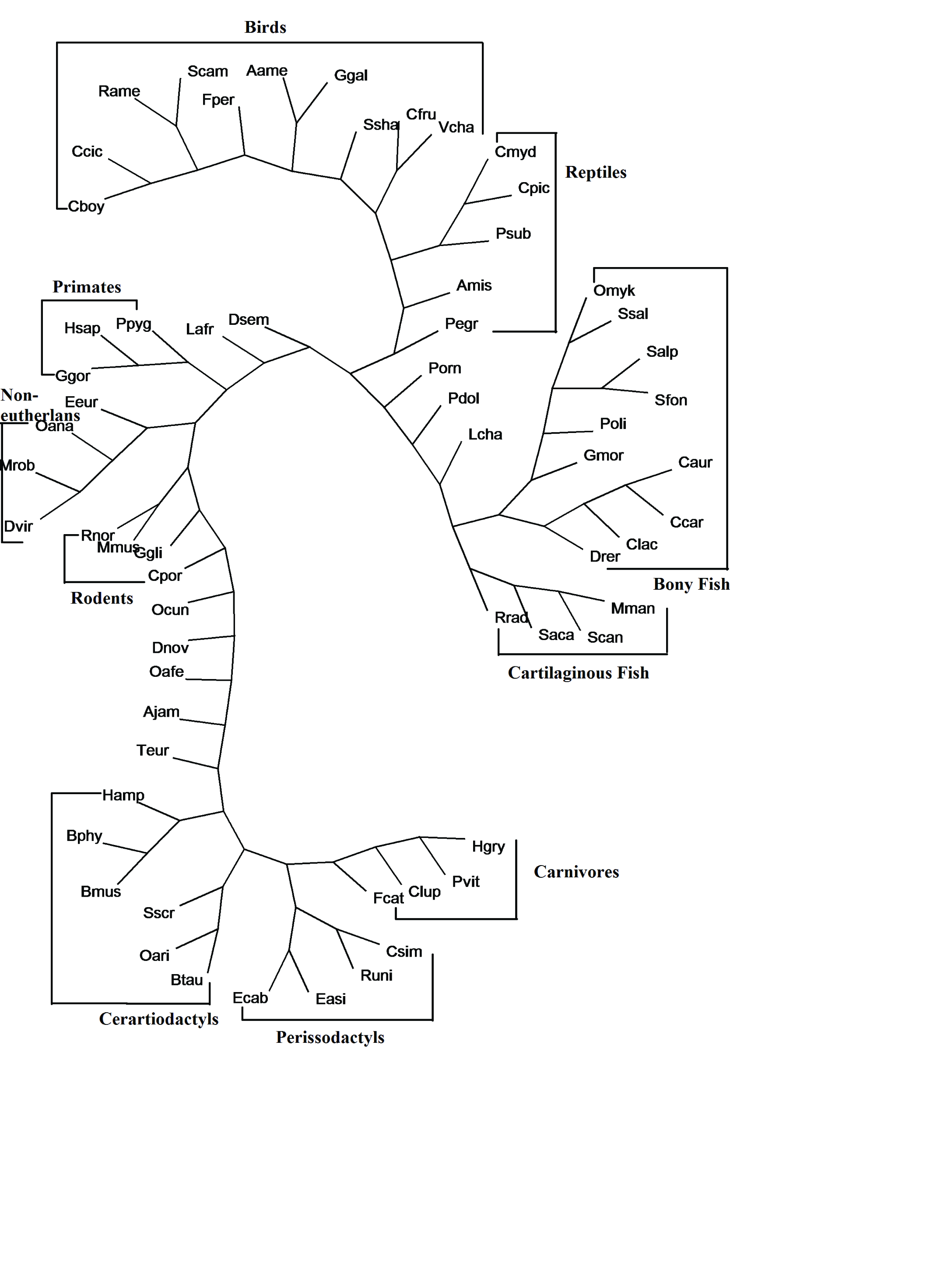

Supplement: Figure S2 — Phylogenetic tree of 64 vertebrate species constructed from 3,055 phylum-specific key K-strings found in Vertebrata. (TIF) [file pone.0084330.s002.tif]

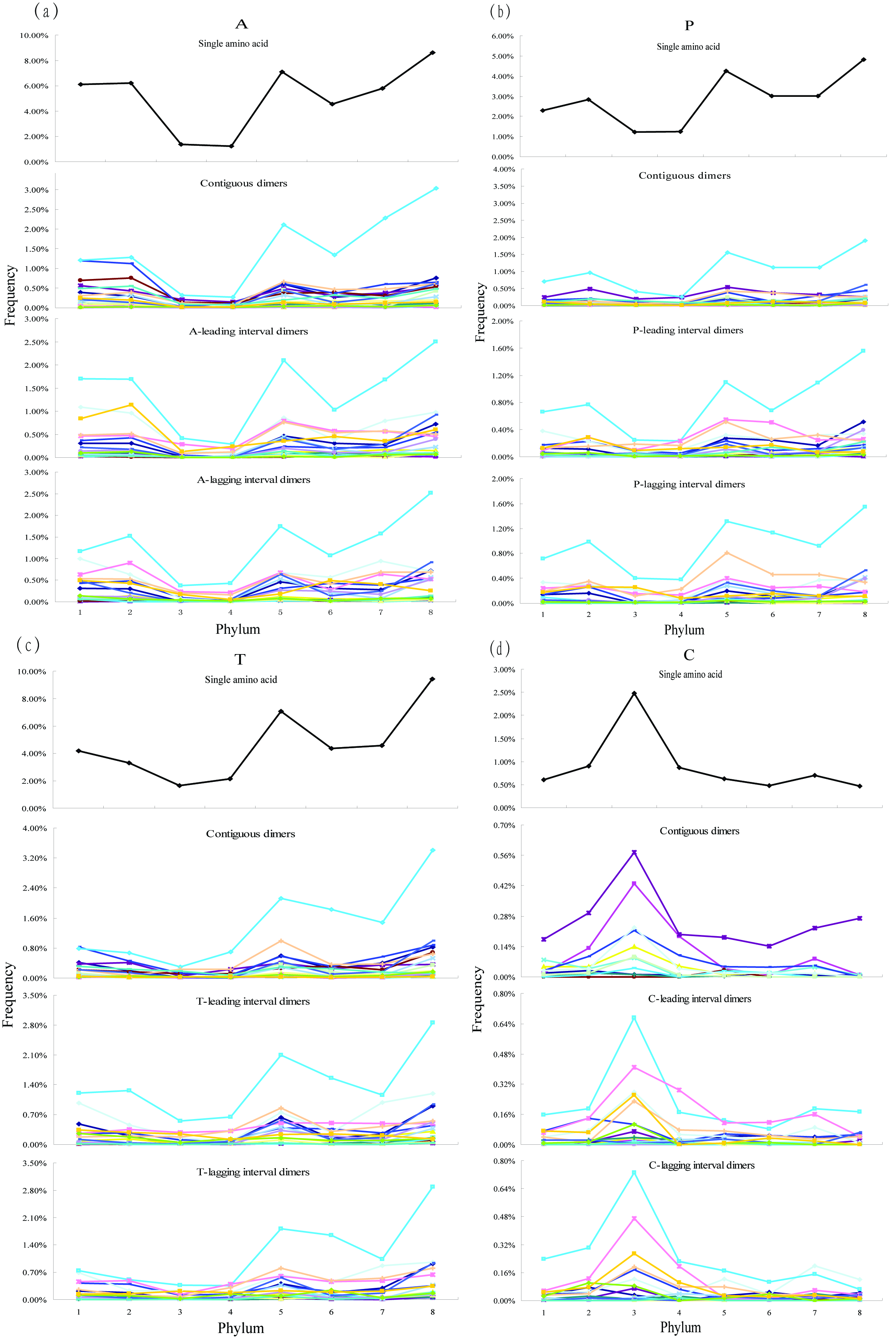

Supplement: Figure S3 — Compositional analysis of phylum-specific key K-strings in 8 phyla. (TIF) [file pone.0084330.s003.tif]
