# Supplementary material for: Phylogenetic and Biological Significance of Evolutionary Elements from Metazoan Mitochondrial Genomes
Source: PLoS One. 2014 Jan 20;9(1):e84330. doi: 10.1371/journal.pone.0084330 (PMC3896360; doi:10.1371/journal.pone.0084330)
Supplement: Table S1 — The dataset of metazoan species for which complete mitochondrial genomes are available. (DOC) [file pone.0084330.s004.doc]

**Table S1. The dataset of species in metazoa whose complete mitochondrial genomes are available.** **^a^**

| **Clade** | **Mitochondrial genomes Number** |
| --- | --- |
| **Metazoa** | **2027** |
| Porifera | 27 |
| Placozoa | 4 |
| Coelenterata | 36 |
| Platyhelminthes | 33 |
| Acanthocephala | 1 |
| Nematoda | 44 |
| Priapulida | 1 |
| Bryozoa | 3 |
| Brachiopoda | 3 |
| Echiura | 2 |
| Mollusca | 83 |
| Annelida | 11 |
| **Arthropoda** | **323** |
| Crustacea | 60 |
| Hexapoda | 207 |
| Chelicerata | 47 |
| Myriapoda | 9 |
| Chaetognatha | 5 |
| Xenoturbellida | 1 |
| Echinodermata | 28 |
| Hemichordata | 3 |
| **Chordata** | **1419** |
| Urochordata | 12 |
| Cephalochordata | 8 |
| Hyperptreti | 2 |
| [Vertebrata](http://www.ncbi.nlm.nih.gov/Taxonomy/Browser/wwwtax.cgi?id=7742&lvl=0) | 1397 |

^a^ As of July 23, 2010, complete mt-genome data were available for 2,027 metazoans at the NCBI web site. We considered 24 phyla (including subphyla); the number of species in each phylum is listed.
