# Supplementary material for: Phylogenetic and Biological Significance of Evolutionary Elements from Metazoan Mitochondrial Genomes
Source: PLoS One. 2014 Jan 20;9(1):e84330. doi: 10.1371/journal.pone.0084330 (PMC3896360; doi:10.1371/journal.pone.0084330)
Supplement: Table S2 — The dataset of 87 species used for tree construction and comparison. (DOC) [file pone.0084330.s005.doc]

**Table S2. The dataset of 87 species used for the tree construction and comparison. ^a^**

|  | **Full name** | **Short name** | | **Full name** | **Short name** |
| --- | --- | --- | --- | --- | --- |
| 1 | *Lubomirskia baicalensis* | Polbai | 45 | *Phlaeoba albonema* | Hepalb |
| 2 | *Agelas schmidti* | Poasch | 46 | *Ostrinia nubilalis* | Heonub |
| 3 | *Ephydatia muelleri* | Poemue | 47 | *Ornithoctonus huwena* | Chohuw |
| 4 | *Axinella corrugata* | Poacor | 48 | *Ixodes persulcatus* | Chiper |
| 5 | *Placozoan sp. BZ49* | Papsp1 | 49 | *Phalangium opilio* | Chpopi |
| 6 | *Placozoan sp. BZ10101* | Papsp2 | 50 | *Buthus occitanus* | Chbocc |
| 7 | *Trichoplax adhaerens* | Patadh | 51 | *Scutigera coleoptrata* | Myscol |
| 8 | *Placozoan sp. BZ2423* | Papsp3 | 52 | *Narceus annularus* | Mynann |
| 9 | *Dendronephthya gigantea* | Codgig | 53 | *Epiperipatus biolleyi* | Myebio |
| 10 | *Seriatopora caliendrum* | Coscal | 54 | *Antrokoreana gracilipes* | Myagra |
| 11 | *Pocillopora damicornis* | Copdam | 55 | *Xenoturbella bocki* | Xexboc |
| 12 | *Montastraea faveolata* | Comfav | 56 | *Balanoglossus clavigerus* | Hmbcla1 |
| 13 | *Taenia solium* | Pttsol | 57 | *Arbacia lixula* | Ecalix |
| 14 | *Taenia hydatigena* | Ptthyd | 58 | *Echinocardium cordatum* | Ececor |
| 15 | *Taenia multiceps* | Pttmul | 59 | *Phanogenia gracilis* | Ecpgra |
| 16 | *Gyrodactylus salaris* | Ptgsal | 60 | *Balanoglossus clavigerus* | Hmbcla2 |
| 17 | *Leptorhynchoides thecatus* | Aclthe | 61 | *Balanoglossus carnosus* | Hmbcar |
| 18 | *Setaria digitata* | Nesdig | 62 | *Saccoglossus kowalevskii* | Hmskow |
| 19 | *Ascaris suum* | Neasuu | 63 | *Lophura nycthemera* | BLnyc |
| 20 | *Romanomermis culicivorax* | Nercul | 64 | *Hemiphaga novaeseelandiae* | Bhnov |
| 21 | *Strelkovimermis spiculatus* | Nesspi | 65 | *Ardea novaehollandiae* | BAnov |
| 22 | *Priapulus caudatus* | Prpcau | 66 | *Syrmaticus humiae* | BShun |
| 23 | *Watersipora subtorquata* | Bywsub | 67 | *Gallirallus okinawae* | BGoki |
| 24 | *Bugula neritina* | Bybner | 68 | *Basiliscus vittatus* | RBvit |
| 25 | *Flustrellidra hispida* | Byfhis | 69 | *Alligator sinensis* | RAsin |
| 26 | *Terebratulina retusa* | Batret | 70 | *Kinyongia fischeri* | RKfis |
| 27 | *Terebratalia transversa* | Battra | 71 | *Acrochordus granulatus* | RAgra |
| 28 | *Laqueus rubellus* | Balrub | 72 | *Eretmochelys imbricata* | RFimb |
| 29 | *Urechis caupo* | Ecucau | 73 | *Uncia uncia* | MUunc |
| 30 | *Urechis unicinctus* | Ecuuni | 74 | *Hemiechinus auritus* | Mhaur |
| 31 | *Meretrix meretrix* | Mommer | 75 | *Vombatus ursinus* | MVurs |
| 32 | *Watasenia scintillans* | Mowsci | 76 | *Nasalis larvatus* | MNlar |
| 33 | *Crassostrea sikamea* | Mocsik | 77 | *Panthera pardus* | MPpar |
| 34 | *Mytilus edulis* | Momedu | 78 | *Bufo japonicus* | ABjap |
| 35 | *Whitmania pigra* | Anwpig | 79 | *Hynobius formosanus* | AHfor |
| 36 | *Platynereis dumerilii* | Anpdum | 80 | *Rhacophorus schlegelii* | ARsch |
| 37 | *Pista cristata* | Anpcri | 81 | *Thorius n. sp.* RLM-2004 | ATnsp |
| 38 | *Orbinia latreillii* | Anolat | 82 | *Ranodon sibiricus* | ARsib |
| 39 | *Metacrangonyx longipes* | Crmlon | 83 | *Lagocephalus lunaris* | FLlun |
| 40 | *Macrobrachium rosenbergii* | Crmros | 84 | *Takifugu xanthopterus* | FTxan |
| 41 | *Scylla tranquebarica* | Crstra | 85 | *Hypentelium nigricans* | FHnig |
| 42 | *Friesea grisea* | Crfgri | 86 | *Polypterus senegalus* | FPsen |
| 43 | *Aleurodicus dugesii* | Headug | 87 | *Distoechodon tumirostris* | FDtum |
| 44 | *Cephus cinctus* | Heccin |  |  |  |

^a^ The full and short names of the 87 selected species are listed. The first two letters of the short name stand for the phylum to which the species belongs: Po, Porifera; Pa, Placozoa; Co, Coelenterata; Pt, Platyhelminthes; Ac, Acanthocephala; Ne, Nematoda; Pr, Priapulida; By, Bryozoa; Ba, Brachiopoda; Ec, Echiura, Echinodermata; Mo, Mollusca; An, Annelida; Cr, Crustacea; He, Hexapoda, Hm, Hemichordata; Ch, Chelicerata; My, Myriapoda; Xe, Xenoturbellida; B, Birds; F, Fishes; A, Amphibians; M, Mammals; R, Reptiles.
